# MoJKNet: a jumping knowledge graph framework for multi-omics cancer subtype prediction

**DOI:** 10.3389/fgene.2026.1803456

**Published:** 2026-04-07

**Authors:** Jiangjie Lou, Xiaoguang Pan, Xuanlong Wang

**Affiliations:** College of Artificial Intelligence and Software, Liaoning Petrochemical University, Fushun, Liaoning, China

**Keywords:** autoencoder, cancer subtype classification, graph attention network, jumping knowledge network, multi-omics integration

## Abstract

Cancer remains one of the leading causes of morbidity and mortality worldwide and poses a major threat to global public health. Despite substantial advances in early diagnosis and therapeutic strategies, patient outcomes vary widely due to the pronounced molecular and clinical heterogeneity of tumors. Accurate identification of cancer subtypes is therefore essential for elucidating tumor heterogeneity, improving prognostic assessment, and enabling precision medicine. In recent years, multi-omics technologies have provided unprecedented opportunities to characterize cancer at multiple molecular layers, including genomic, epigenomic, transcriptomic, and proteomic levels. However, effectively integrating high-dimensional and heterogeneous multi-omics data remains a major challenge. Moreover, many existing graph convolutional network–based integration methods suffer from over-smoothing and limited utilization of deep feature representations, which restrict their ability to capture complex multi-scale relationships inherent in cancer biology. To address these challenges, we propose MoJKNet, a novel multi-omics integration framework for cancer subtype classification. MoJKNet incorporates a jumping knowledge network (JK-Net) to adaptively aggregate node representations across multiple propagation depths, thereby alleviating over-smoothing and enhancing feature extraction within each omics modality. Subsequently, a multimodal autoencoder combined with similarity network fusion (SNF) is employed to capture complementary information across different omics layers. Finally, a graph attention network (GAT) assigns adaptive feature weights to enable accurate cancer subtype prediction. We evaluated MoJKNet on seven cancer types from The Cancer Genome Atlas (TCGA). Experimental results demonstrate that MoJKNet consistently outperforms state-of-the-art methods, including MOGCAN, MOGONET, and MoGCN, in terms of precision, recall, and F1-score, achieving nearly a 10% performance improvement on the COADREAD dataset. Ablation studies further confirm the critical contribution of the jumping knowledge mechanism to improved representation learning. Overall, MoJKNet provides an effective and generalizable solution for multi-omics data integration and cancer subtype classification, with strong potential for downstream biological interpretation and translational applications.

## 1 Introduction

Cancer is a highly heterogeneous and complex disease (Subramanian et al., 2020). Although precision medicine has achieved notable advances in cancer prevention, diagnosis, and treatment, its overall clinical effectiveness remains limited (Lu and Zhan, 2018). In recent years, multi-omics analytical strategies have provided new opportunities to elucidate the molecular mechanisms underlying disease. By integrating multiple omics datasets derived from the same patient (Heo et al., 2021), researchers can obtain a comprehensive view of disease initiation and progression across distinct biological layers. This integrative paradigm has been successfully applied in studies of Alzheimer’s disease (Ginn et al., 2025), Parkinson’s disease (Chan et al., 2022; Wang et al., 2022), and various cancer types. Consequently, an increasing number of studies have begun to systematically characterize the molecular and clinical heterogeneity of cancer from a multi-omics perspective (Sun and Hu, 2016).

With the rapid development of high-throughput sequencing technologies, large-scale multi-omics datasets have been widely used for cancer subtype prediction and patient stratification. Early studies, such as those by Ye et al. (2023) and Raj et al. (2024), applied principal component analysis (PCA) for dimensionality reduction. However, although unsupervised feature learning methods are commonly employed, they are inherently limited by their inability to effectively distinguish noise from task-relevant features. To address this limitation, graph-based deep learning methods have been increasingly adopted. Li et al. (2021) introduced a graph convolutional network (GCN) framework (Kipf and Welling, 2017) that integrates gene expression and copy number alteration data with multiple prior knowledge graphs, including gene–gene interaction (GGI), protein–protein interaction (PPI), and gene co-expression networks, to classify 28 pan-cancer types. Zhou et al. (2022) integrated gene expression, DNA methylation, and miRNA expression data by constructing sample similarity networks and applied a graph convolutional autoencoder to identify novel cancer subtypes across breast, brain, colon, and kidney cancers. Guo et al. (2023) further incorporated PPI network topology with multi-omics features and introduced an attention mechanism on top of GCN embeddings for breast cancer subtype classification.

More recently, several advanced multi-omics integration frameworks have been proposed. Li et al. developed the MoGCN model (Li et al., 2022), which employs autoencoders for feature extraction and similarity network fusion (SNF) (Wang et al., 2014) to construct patient similarity networks, followed by GCN-based subtype classification for breast and pan-kidney cancers. MOGONET (Wang et al., 2021) utilizes GCNs to learn omics-specific representations and constructs a cross-omics feature tensor to capture shared and complementary information, which is subsequently integrated through a view correlation discovery network (VCDN). The SUPREME model (Nesibe Kesimoglu and Bozdag, 2023) integrates seven data modalities—including gene expression, miRNA expression, DNA methylation, single nucleotide variation, copy number alteration, co-expression module eigengenes, and clinical data—and combines GCNs with a multilayer perceptron (MLP) for breast cancer subtype analysis. MO-GCAN (Dou and Mirzaei, 2025) learns omics-specific representations using GCNs and refines them via a graph attention network (GAT) (Velickovic et al., 2017) to perform adaptive feature weighting. In contrast, MOGAT (Tanvir et al., 2024) integrates eight data modalities and employs GATs to learn omics-specific latent representations, followed by MLP-based classification.

Despite these advances, most existing cancer subtype prediction models rely on fixed-depth GCNs or GATs for omics feature extraction. Traditional GCNs are prone to over-smoothing when stacked into deeper architectures, while GATs, although introducing attention mechanisms to enhance neighborhood selectivity, remain constrained by their fixed propagation depth. Jumping knowledge networks (JK-Net) (Xu et al., 2018) address this limitation by flexibly aggregating node representations from multiple propagation layers, thereby enabling the capture of complex nonlinear dependencies while effectively mitigating the over-smoothing problem.

**FIGURE 1:**
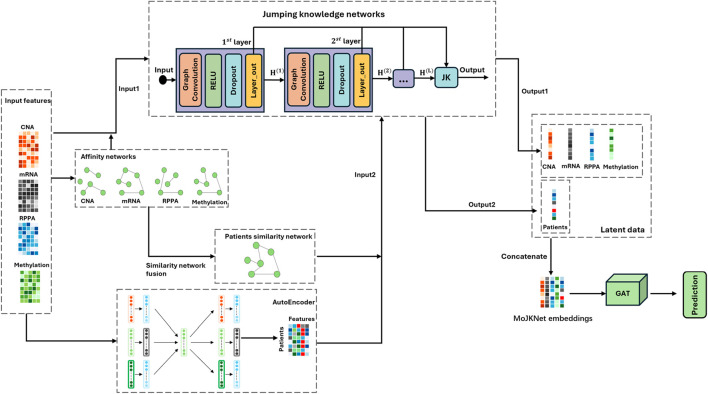
Schematic overview of the MoJKNet model.

In this study, we propose MoJKNet, a novel multi-omics cancer subtype classification framework that incorporates jumping knowledge connections, as illustrated in Figure 1. The proposed model aims to learn both intra-omics and inter-omics representations by extracting latent features from individual omics modalities as well as integrated multi-omics embeddings, thereby jointly modeling omics-specific and cross-omics knowledge graphs (Ji et al., 2022). Extensive experiments were conducted on datasets from seven cancer types, including subtype classification (Zhao et al., 2022), subtype visualization, and survival analysis, to comprehensively evaluate the effectiveness and robustness of the proposed framework.

## 2 Materials and methods

### 2.1 Dataset preparation and preprocessing

The multi-omics datasets used in this study were obtained from the cBioPortal for Cancer Genomics (https://www.cbioportal.org/) (Cerami et al., 2012), which provides curated and harmonized data derived from The Cancer Genome Atlas (TCGA) Pan-Cancer Atlas. We collected four types of omics data, including copy number alteration (CNA) (Mirzaei and Petreaca, 2022), DNA methylation, mRNA sequencing (mRNA), and reverse phase protein array (RPPA) data. All datasets were downloaded from cBioPortal in 2024.

The datasets cover seven cancer types: TCGA-UCEC (uterine corpus endometrial carcinoma), TCGA-STAD (stomach adenocarcinoma), TCGA-SARC (sarcoma), TCGA-COADREAD (colorectal adenocarcinoma), TCGA-LGG (lower-grade glioma), TCGA-HNSC (head and neck squamous cell carcinoma), and TCGA-BRCA (breast invasive carcinoma). Detailed sample distributions for each cancer type are summarized in Table 1.

**TABLE 1:** The multi-omics data scales and label distributions of the seven cancer types.

Cancer datesets	CNA	Methylation	mRNA	RPPA	Classes	Class distribution
TCGA-UCEC	17913	22599	17508	192	4	(128, 119, 116, 42)
TCGA-STAD	24481	22583	16766	194	5	(178, 62, 39, 26, 6)
TCGA-SARC	24752	22539	20155	194	4	(75, 63, 42, 14)
TCGA-COADREAD	23331	22585	17508	192	8	(175, 77, 49, 45, 7, 5, 3, 2)
TCGA-LGG	19453	22589	20164	191	3	(204, 145, 72)
TCGA-HNSC	25129	22587	20224	192	2	(186, 16)
TCGA-BRCA	23706	22593	20212	191	5	(381, 167, 141, 71, 27)

For all datasets except TCGA-BRCA, every class represents a distinct cancer subtype (e.g., molecular). Only in the TCGA-BRCA (Breast Cancer) dataset, the first four classes (381, 167, 141, 71) are cancer subtypes, while the fifth class (27 samples) consists of normal tissue samples.

To prevent inconsistencies arising from duplicated entries and missing subtype labels, we excluded all samples without SUBTYPE information in the clinical dataset. During preprocessing of each omics dataset, samples lacking subtype annotations were also removed. Duplicate samples across datasets were identified and eliminated, retaining only the last occurrence. To ensure consistent mapping between omics files and clini-cal records, all sample identifiers were truncated to the first 12 characters (e.g., “TCGA-CS-4938-01” was converted to “TCGA-CS-4938”). Missing values were observed both within individual omics layers and across omics types (Song et al., 2020). For with-in-omics missingness, features with >10% missing values were discarded, and the re-maining missing entries were imputed with zero. We further removed features in which >10% of samples had a value of zero, as these features typically exhibit low variability and provide limited discriminatory information. For cross-omics missingness, samples lacking any of the four omics modalities were excluded to ensure alignment across datasets. Each retained sample comprised five files: CNA, DNA methylation, mRNA expression, RPPA, and subtype. Finally, all datasets were sorted by sample ID to maintain a consistent sample order across omics type.

For all algorithms, five-fold cross-validation was employed to evaluate model performance. Specifically, the dataset containing cancer subtype labels was randomly divided into five subsets. In each iteration, one subset was used as the test set, while the remaining four subsets were used for model training, resulting in five different training–testing splits. In each run, the model was trained on the training set and evaluated on the corresponding test set. The final performance was reported as the average of the evaluation metrics across the five runs, ensuring the stability and reliability of the experimental results.

#### 2.1.1 Affinity network construction

For each omics modality, an independent affinity network was constructed to characterize patient–patient similarity based on molecular profiles. Specifically, for a given omics type m , each patient was represented by a feature vector $x_{i}^{\left(m\right)}$ . Pairwise similarities between patients i and j were computed using a Gaussian kernel function:

$$s_{ij}^{\left(m\right)}=\exp\left(-\frac{∥x_{i}^{\left(m\right)}-x_{j}^{\left(m\right)}{∥}_{2}^{2}}{\sigma_{m}^{2}}\right)$$

where $∥\cdot {∥}_{2}$ denotes the Euclidean distance and $\sigma_{m}$ is a scaling parameter specific to each omics dataset.

To preserve local neighborhood structures and reduce the influence of noise, the similarity matrix was sparsified using a *k*-nearest neighbor (k-NN) strategy. For each patient, only similarities with the top-*k* most similar neighbors were retained, resulting in a weighted affinity matrix for each omics modality. These affinity networks capture complementary aspects of patient similarity across different molecular layers.

#### 2.1.2 Similarity network fusion

The omics-specific affinity networks were integrated using the similarity network fusion (SNF) algorithm to generate a single fused patient similarity network. SNF iteratively updates each affinity network by incorporating information from the remaining networks, allowing shared similarity patterns to be reinforced while preserving modality-specific information. At each iteration, the similarity structure of one omics network was updated based on the normalized similarity matrices of the other omics networks. This process was repeated for a predefined number of iterations until convergence. The final fused network represents a consensus patient similarity graph that integrates CNA, DNA methylation, mRNA expression, and RPPA data. The resulting SNF-based patient similarity network provides a comprehensive representation of inter-patient relationships and serves as a robust foundation for downstream analyses, such as graph-based feature learning and clinical outcome prediction.

### 2.2 The classification model based on multi-omics data integration

In this study, we employ the MoJKNet model to classify seven cancer subtypes based on multi-omics data. The model consists of affinity networks, patient similarity networks, and a jump knowledge network. For this problem, MoJKNet constructs affinity networks from four omics modalities (CNA, mRNA, RPPA, and Methylation) and performs representation learning through two input pathways. As illustrated in Figure 1, Input1 uses raw omics features together with their corresponding affinity networks, while Input2 integrates patient similarity networks with latent features learned by an autoencoder. Jumping knowledge network (JK-Net) are applied to both pathways to generate two sets of latent representations (Latent data), referred to as Output1 and Output2. Latent data are then concatenated to form the final MoJKNet embeddings, which is subsequently used for cancer subtype prediction, visualization, and survival analysis.

#### 2.2.1 Multimodal autoencoder

The multimodal autoencoder is an unsupervised neural network composed of an encoder and a decoder. The encoder maps the input data to a latent space representation $h=f\left(x\right)$ , while the decoder reconstructs the original input from the latent representation, $\hat{x}=g\left(h\right)$ . By minimizing the reconstruction loss, the model captures the key features of the data. The loss function is defined as:

$$L=\mathrm{argmin}\left[\text{Loss}\left(x,\hat{g}\left(x\right)\right)\right]$$

Since the input consists of multi-omics data types represented by multiple feature matrices $X_{1},X_{2},X_{3},X_{4}$ , which include DNA copy number alterations, methylation data, transcriptomic data, and proteomic matrix data, the model includes multiple encoders and decoders with the same latent layer, as shown in Figure 1. The loss function is defined as:

$$L=\mathrm{argmin}\left[\alpha {\text{Loss}}_{1}\left(x_{1},{\hat{g}}_{1}\left(x_{1}\right)\right)+\beta {\text{Loss}}_{2}\left(x_{2},{\hat{g}}_{2}\left(x_{2}\right)\right)+\ldots \right]$$

Here, α=0.2,β=0.2,γ=0.3,δ=0.3 represent the weights corresponding to each omics data type (based on prior knowledge), and their sum is constrained to 1 (i.e., α+β+γ+δ=1 ).

#### 2.2.2 Learning features by jump knowledge network

In the MoJKNet model, we adopt a JK-Net to fuse patient representations derived from multiple graph convolutional layers. Each layer encodes patient similarity patterns at a specific neighborhood range within the patient similarity network. By aggregating representations from different layers, the JK-Net enables adaptive integration of both short-range and long-range patient relationships, which is particularly beneficial for modeling heterogeneous interactions across multi-omics data. This design effectively mitigates over-smoothing and preserves discriminative information critical for accurate cancer subtype classification. The node representation at layer *l* is defined as:

$${h_{v}}^{\left(l\right)}=\sigma \left(W^{\left(l\right)}\cdot AGGREGATE\left(\left\{{h_{u}}^{\left(l-1\right)},\forall u\in \tilde{N}\left(v\right)\right\}\right)\right)$$

where $h_{v}^{\left(l\right)}$ is the hidden state of node v at layer l ; $\tilde{N}\left(v\right)$ denotes the neighborhood of v , including the node itself; *AGGREGATE* is the neighborhood aggregation function; $W^{\left(l\right)}$ is the trainable weight matrix of layer l ; and σ is a nonlinear activation function.

Given a total of L layers, JK-Net aggregates representations from layer 0 to layer L to produce a unified final representation:

$${h_{v}}^{\text{final}}=\text{JK}\_\text{AGGREGATE}\left(\left\{h_{v}\left(0\right),h_{v}\left(1\right),...,h_{v}\left(L\right)\right\}\right)$$

Among the commonly used JK_AGGREGATE strategies, we adopt the concatenation approach to combine features from all layers, thereby retaining multi-scale structural information. The final representation obtained via concatenation is expressed as:

Concatenation

$$h_{v}^{\text{final}}=h_{v}^{\left(0\right)}∥h_{v}^{\left(1\right)}∥h_{v}^{\left(2\right)}∥\cdots ∥h_{v}^{\left(L\right)}$$

Max-pooling

$${h_{v}}^{\text{final}}=\max\left({h_{v}}^{\left(0\right)},{h_{v}}^{2},\ldots ,{h_{v}}^{L}\right)$$

#### 2.2.3 Cancer subtype classification by graph attention network

Based on the traditional graph attention network (GAT) (Velickovic et al., 2017), we constructed a GAT model composed of graph attention layers and linear layers for multi-omics data integration. The representations learned by JK-Net were concatenated to form the input feature matrix, whereas the fused network derived from the similarity networks was employed as the graph structure.

#### 2.2.4 Baseline methods and fair comparison settings

For fair and reproducible comparison, all baseline methods were evaluated under the same experimental settings as MoJKNet. Specifically, all methods used the same datasets, identical preprocessing procedures, and the same stratified train–test split (75% training and 25% testing). Performance was evaluated using macro-averaged precision, recall, and F1-score across all datasets. Baseline methods including MoGCN, MOGONET, and MO-GCAN were implemented using the official code released by the original authors. When available, default hyperparameter settings reported in the original papers were adopted. For methods requiring parameter tuning, hyperparameters were selected based on validation performance within the training set only, without using any test data. No method was granted access to additional data or supervision beyond the defined training split.

#### 2.2.5 Implementation details and hyperparameter settings

All hyperparameters are summarized in Tables 2–4.

**TABLE 2:** Hyperparameter tuning for input1 and input of JKNet.

Hyperparameters	Value
JKNET hidden layer dimensions	[100]
JKNET #of epochs	[150]
JKNET learning rate	[0.1, **0.001**, 0.0001]
JKNET dropout	[0.5]
JKNET mode	[**Cat**, max]
Input1 of JKNET layers	[**2(else)**, **3(coadread)**, 4]
Input2 of JKNET layers	[**2(else)**, 3, **4(coadread)**]

Bold values indicate the selected optimal hyperparameters. “else” denotes datasets other than TCGA-COADREAD. “coadread” refers specifically to the TCGA-COADREAD dataset.

**TABLE 3:** Hyperparameter tuning for GAT.

Hyperparameters	Values
GAT hidden layer dimensions	[100]
GAT #of epochs	[150]
GAT learning rate	[0.1, **0.001**, 0.0001]
GAT dropout	[0.5]
GAT # of heads	[1, **2**, 4, 8]
GAT # of alpha	[0.2]

Bold values indicate the selected optimal hyperparameters.

**TABLE 4:** Hyperparameter tuning for reproducibility.

Hyperparameters	Values
Metric for distance calculation	Sqeuclidean
Number of nearest neighbors (K)	20
Fusion parameter	μ = 0.5
Data normalization	float64

### 2.3 Survival analysis

To evaluate the prognostic risk stratification performance of the proposed model, survival analyses were performed based on the risk scores predicted by the model. Patients were divided into high-risk and low-risk groups according to the median value of the risk score.

Overall survival (OS) curves for the two groups were estimated using the kaplan–meier (KM) method, which accounts for follow-up time and right-censoring information. Survival differences between the high-risk and low-risk groups were visualized using KM curves.

The statistical significance of differences between the survival curves was assessed using the Log-rank test. Under the null hypothesis that the two groups share identical survival functions, the Log-rank test compares the cumulative number of observed and expected events over the entire follow-up period. The Log-rank test statistic is defined as:

$$\chi^{2}=\frac{{\left(O_{1}-E_{1}\right)}^{2}}{V_{1}}$$

where $O_{1}$ represents the observed number of events in the high-risk group, $E_{1}$ denotes the expected number of events under the null hypothesis, and $V_{1}$ is the corresponding variance. A two-sided p-value <0.05 was considered statistically significant.

In addition, the concordance index (C-index) was calculated to evaluate the predictive accuracy of the model for survival outcomes. The C-index measures the concordance between the predicted risk scores and the observed survival times and is defined as:

$$C-\text{index}=\frac{\;\text{concordant}\;\text{pairs}}{\text{All}\;\text{comparable}\;\text{pairs}}$$

## 3 Results

### 3.1 Comparison of performance

To evaluate the performance of the proposed MoJKNet framework, we conducted a comprehensive comparison with three state-of-the-art graph neural network–based multi-omics integration methods for cancer subtype prediction, namely MO-GCAN, MOGONET, and MoGCN. These approaches represent leading strategies for multi-omics data integration using graph neural networks and therefore serve as strong baselines for assessing the effectiveness of MoJKNet.

We adopted macro-average (avg) precision, macro-average recall, and macro-average F1-score as the primary evaluation metrics to ensure a balanced performance assessment across cancer subtypes with varying sample sizes. These metrics are less sensitive to class imbalance and are widely used in multi-omics–based cancer subtype prediction tasks. The detailed results for macro-average precision, recall, and F1-score are reported in Tables 5–7, respectively. Overall, MoJKNet consistently outperforms the competing methods across most cancer types, indicating its robustness and enhanced ability to capture complementary information across different omics layers.

**TABLE 5:** Performance comparison (Macro avg precision, %).

Cancer dataset	MO-GCAN	MOGONET	MoGCN	MoJKNet
TCGA-UCEC	75.67 ± 4.56	63.37 ± 4.51	64.93 ± 4.81	**77.43 ± 4.68**
TCGA-STAD	63.85 ± 5.15	63.27 ± 3.74	63.54 ± 5.99	**64.44 ± 4.79**
TCGA-SARC	76.76 ± 10.11	77.33 ± 5.11	76.64 ± 4.49	**81.01 ± 3.67**
TCGA-COADREAD	31.97 ± 2.08	26.89 ± 4.09	27.33 ± 5.82	**34.38 ± 3.98**
TCGA-LGG	**96.86 ± 1.40**	96.56 ± 1.25	96.28 ± 1.18	96.59 ± 2.04
TCGA-HNSC	71.88 ± 9.02	55.98 ± 9.71	56.28 ± 13.01	**97.46 ± 0.87**
TCGA-BRCA	69.92 ± 2.49	70.01 ± 6.53	64.87 ± 2.56	**76.54 ± 5.28**

Bold values indicate the best performance among all compared methods for each cancer dataset. Results are reported as mean ± standard deviation over five-fold cross-validation.

**TABLE 6:** Performance comparison (Macro avg recall, %).

Cancer dataset	MO-GCAN	MOGONET	MoGCN	MoJKNet
TCGA-UCEC	74.16 ± 2.79	61.00 ± 2.34	61.73 ± 1.40	**77.59 ± 4.82**
TCGA-STAD	63.29 ± 4.58	61.65 ± 5.06	60.61 ± 5.36	**65.18 ± 5.15**
TCGA-SARC	74.29 ± 6.28	78.79 ± 5.00	76.75 ± 7.92	**82.87 ± 3.54**
TCGA-COADREAD	37.59 ± 2.78	26.53 ± 5.19	30.23 ± 1.90	**37.76 ± 3.90**
TCGA-LGG	**97.98 ± 1.35**	95.22 ± 2.77	95.07 ± 2.97	96.93 ± 1.90
TCGA-HNSC	84.67 ± 3.53	54.68 ± 6.37	53.33 ± 7.45	**88.95 ± 9.84**
TCGA-BRCA	67.26 ± 3.54	69.63 ± 4.37	58.47 ± 3.27	**73.37 ± 4.26**

Bold values indicate the best performance among all compared methods for each cancer dataset. Results are reported as mean ± standard deviation over five-fold cross-validation.

**TABLE 7:** Performance comparison (Macro avg F1-score, %).

Cancer dataset	MO-GCAN	MOGONET	MoGCN	MoJKNet
TCGA-UCEC	74.11 ± 3.38	58.84 ± 2.98	60.43 ± 2.35	**77.05 ± 4.71**
TCGA-STAD	64.07 ± 4.27	61.39 ± 4.28	61.65 ± 5.06	**64.21 ± 4.51**
TCGA-SARC	72.56 ± 6.19	73.42 ± 3.73	75.86 ± 6.28	**79.96 ± 1.75**
TCGA-COADREAD	34.93 ± 1.61	23.90 ± 3.00	26.18 ± 2.31	**34.66 ± 4.07**
TCGA-LGG	**97.35 ± 1.33**	96.56 ± 1.97	95.98 ± 2.17	96.58 ± 1.70
TCGA-HNSC	73.25 ± 9.52	52.45 ± 9.47	53.07 ± 11.53	**88.30 ± 8.90**
TCGA-BRCA	67.17 ± 5.37	70.41 ± 5.35	59.78 ± 3.20	**73.53 ± 4.44**

Bold values indicate the best performance among all compared methods for each cancer dataset. Results are reported as mean ± standard deviation over five-fold cross-validation.

#### 3.1.1 Omics-specific contribution in prediction

To further investigate the contribution of latent representations to cancer subtype prediction, we evaluated five types of latent data across seven cancer datasets, as summarized in Table 1. The latent data considered include mRNA expression (mRNA), DNA methylation (MET), copy number alteration (CNA), protein expression (RPPA), and patient-level data (Patients). The latent representations for mRNA, MET, CNA, and RPPA were directly generated using the jumping knowledge network (JK-Net), whereas the patient-level representation was obtained through a joint framework combining an autoencoder and JK-Net, which integrates information learned from the four primary omics modalities. The last column (ALL) reports the performance achieved by concatenating latent representations from all data types.

The results reveal that the predictive contribution of each omics-derived latent representation varies considerably across cancer types, as summarized in Table 8. Among the individual modalities, mRNA representations consistently achieve strong performance across multiple datasets, particularly in UCEC, SARC, BRCA, and LGG, where they obtain the highest or near-highest macro-average scores among single-omics inputs. This finding suggests that transcriptomic information plays a critical role in subtype discrimination for these cancers. For MET, competitive performance is observed in several datasets, especially STAD and LGG, indicating that methylation patterns provide valuable complementary information for subtype identification. In contrast, the predictive ability of CNA is relatively limited in most datasets, although moderate performance can still be observed in cancers such as HNSC and LGG. Similarly, RPPA generally shows moderate predictive capability across datasets, reflecting that protein expression features alone may not always provide sufficient discriminative information for subtype classification. The patient-level representations, which capture integrated features learned from multiple omics modalities through the autoencoder–JK-Net framework, achieve relatively strong performance in several datasets, including STAD, COADREAD, and LGG, demonstrating the effectiveness of higher-level feature integration in capturing complementary biological signals.

**TABLE 8:** Contribution of each omics data type. This table reports macro average (avg) precision, macro avg recall, and macro avg F1-score of MoJKNet across seven cancer datasets under different omics combinations. Each row shows the model performance when excluding the omics type listed in the first column, enabling assessment of its individual contribution. The final row presents the results obtained when integrating all omics data types.

Multi-class metrics	Cancer dataset	CNA	MET	mRNA	RPPA	Patients	All
Macro avg precision	UCEC	50.42 ± 3.52	65.90 ± 8.10	78.08 ± 3.75	67.29 ± 4.49	66.68 ± 9.79	**77.43 ± 4.68**
	STAD	37.80 ± 11.53	67.97 ± 4.24	62.27 ± 6.06	49.27 ± 7.52	64.77 ± 6.33	**64.44 ± 4.79**
	SARC	51.57 ± 8.53	82.67 ± 6.69	85.06 ± 6.30	75.03 ± 5.32	77.28 ± 4.25	**81.01 ± 3.67**
	COADREAD	26.40 ± 6.91	29.32 ± 4.85	30.77 ± 6.20	26.07 ± 7.23	31.81 ± 5.44	**34.38 ± 3.98**
	LGG	90.98 ± 6.53	96.48 ± 1.43	96.55 ± 2.41	78.61 ± 4.63	96.60 ± 1.98	**96.59 ± 2.04**
	HNSC	87.43 ± 11.77	88.47 ± 12.09	87.91 ± 14.20	72.77 ± 13.67	86.00 ± 14.55	**97.46 ± 0.87**
	BRCA	54.45 ± 3.73	46.99 ± 8.91	86.01 ± 3.77	59.84 ± 3.10	65.81 ± 3.89	76.54 ± 5.28
Macro avg recall	UCEC	53.21 ± 2.84	65.34 ± 4.01	81.05 ± 4.62	66.09 ± 3.22	63.37 ± 3.06	77.59 ± 4.82
	STAD	38.20 ± 10.67	62.53 ± 4.05	61.57 ± 4.92	50.06 ± 3.07	62.49 ± 4.29	**65.18 ± 5.15**
	SARC	52.79 ± 8.78	78.52 ± 4.80	84.84 ± 4.91	72.91 ± 3.58	74.65 ± 11.06	**82.87 ± 3.54**
	COADREAD	28.24 ± 6.99	31.55 ± 4.32	36.60 ± 3.27	20.66 ± 5.76	32.13 ± 3.11	**37.76 ± 3.90**
	LGG	88.69 ± 5.26	94.17 ± 2.16	94.80 ± 2.67	76.44 ± 7.06	94.91 ± 3.08	**96.93 ± 1.90**
	HNSC	80.56 ± 8.71	69.17 ± 18.54	73.63 ± 10.88	80.74 ± 13.10	69.17 ± 8.12	**88.95 ± 9.84**
	BRCA	53.21 ± 2.57	43.76 ± 5.90	81.37 ± 1.63	58.52 ± 3.41	59.43 ± 4.88	73.37 ± 4.26
Macro avg F1-score	UCEC	49.99 ± 3.09	64.13 ± 5.45	80.31 ± 4.15	66.10 ± 3.18	62.50 ± 4.04	**77.05 ± 4.71**
	STAD	35.72 ± 9.50	62.22 ± 4.34	62.38 ± 5.46	48.93 ± 4.93	62.84 ± 4.00	**64.21 ± 4.51**
	SARC	51.14 ± 8.07	78.85 ± 3.97	82.80 ± 7.49	72.91 ± 3.58	74.23 ± 9.47	79.96 ± 1.75
	COADREAD	25.84 ± 7.06	29.09 ± 4.32	32.58 ± 4.14	30.66 ± 5.76	29.48 ± 3.80	**34.66 ± 4.07**
	LGG	89.45 ± 5.80	95.58 ± 2.75	95.62 ± 2.55	76.22 ± 5.33	95.68 ± 2.46	**96.58 ± 1.70**
	HNSC	85.64 ± 9.94	72.69 ± 18.55	78.25 ± 11.71	70.25 ± 5.18	75.69 ± 8.02	**88.30 ± 8.90**
	BRCA	53.24 ± 2.70	41.22 ± 5.46	81.49 ± 2.07	58.57 ± 2.85	60.59 ± 4.81	73.53 ± 4.44

Red: Highest performance among all combinations excluding a single omics type. Blue: Lowest performance among all combinations excluding a single omics type. Bold: Best overall performance when all omics modalities are included.

Overall, the results demonstrate that all latent data types contribute to cancer subtype prediction; however, their relative importance varies substantially across cancer types. This observation underscores the necessity of multi-omics integration for achieving comprehensive and accurate subtype prediction.

#### 3.1.2 Ablation study

To further assess the effectiveness of the jumping knowledge network (JK-Net) within the MoJKNet framework, we conducted ablation experiments comparing JK-Net with a standard graph convolutional network (GCN). As illustrated in the Input1 and Input2 branches in Figure 1, JK-Net employs multiple graph convolution layers to model each omics modality and aggregates node representations from different propagation depths through a jumping knowledge mechanism. This ablation study aims to evaluate whether JK-Net can more effectively exploit multi-omics information and improve classification performance compared with conventional GCNs.

Under identical experimental settings, JK-Net and GCN were evaluated on seven TCGA multi-omics cancer datasets to assess the effectiveness of the jumping knowledge mechanism. The results for the Input1 branch (Table 9) show that JK-Net outperforms GCN on five of the seven datasets, with an average improvement of approximately 6.14 percentage points. Particularly notable improvements are observed for TCGA-HNSC and TCGA-COADREAD, where JK-Net achieves substantial performance gains, suggesting that the jumping knowledge mechanism effectively alleviates the over-smoothing problem commonly encountered in deeper GCN architectures by preserving informative representations from different neighborhood ranges. On TCGA-LGG and TCGA-BRCA, GCN slightly outperforms JK-Net, which may be attributed to dataset-specific graph characteristics where shallow feature propagation is sufficient to capture the underlying structure.

**TABLE 9:** Macro avg precision of comparing JK-Net and GCN on the Input1 branch across seven TCGA multi-omics cancer datasets.

Cancer datesets	JK-net (%)	GCN (%)
TCGA-UCEC	**77.43 ± 4.68**	75.78 ± 3.42
TCGA-STAD	**64.44 ± 4.79**	63.07 ± 6.68
TCGA-SARC	**81.01 ± 3.67**	80.95 ± 3.23
TCGA-COADREAD	**34.38 ± 3.98**	28.27 ± 6.87
TCGA-LGG	96.59 ± 2.04	**97.30 ± 1.83**
TCGA-HNSC	**97.46 ± 0.87**	77.67 ± 5.42
TCGA-BRCA	76.54 ± 5.28	**79.16 ± 4.45**

Bold values indicate the best performance achieved by the full model or its variants in each evaluation setting. Results are reported as mean ± standard deviation over five-fold cross-validation.

For the Input2 branch (Table 10), JK-Net demonstrates improved performance on four of the seven datasets and maintains competitive results on the remaining datasets, with an average improvement of approximately 4.41 percentage points. Significant gains are observed for TCGA-HNSC and TCGA-COADREAD, indicating that JK-Net can more effectively integrate multi-hop neighborhood information while suppressing noise propagation in patient similarity graphs. In addition, JK-Net achieves modest improvements on TCGA-UCEC, TCGA-STAD, and TCGA-BRCA, while GCN performs slightly better on TCGA-SARC and TCGA-LGG, suggesting that the effectiveness of deeper representation aggregation may vary depending on the structural properties of the patient similarity graph.

**TABLE 10:** Macro avg precision of comparing JK-Net and GCN on the Input2 branch across seven TCGA multi-omics cancer datasets.

Cancer datesets	JK-net (%)	GCN (%)
TCGA-UCEC	**77.43 ± 4.68**	76.60 ± 5.35
TCGA-STAD	**64.44 ± 4.79**	62.26 ± 4.64
TCGA-SARC	**81.01 ± 3.67**	81.30 ± 3.08
TCGA-COADREAD	**34.38 ± 3.98**	29.23 ± 2.99
TCGA-LGG	96.59 ± 2.04	**97.49 ± 0.87**
TCGA-HNSC	**97.46 ± 0.87**	78.95 ± 13.55
TCGA-BRCA	**76.54 ± 5.28**	75.85 ± 5.16

Bold values indicate the best performance achieved by the full model or its variants in each evaluation setting. Results are reported as mean ± standard deviation over five-fold cross-validation.

Notably, the performance gains introduced by JK-Net are not uniform across different omics modalities. High-dimensional omics data such as mRNA expression tend to benefit more substantially from the jumping knowledge mechanism, as transcriptomic data encode complex regulatory relationships where discriminative patterns may exist at multiple neighborhood scales. By adaptively aggregating representations from both shallow and deep graph convolutional layers, JK-Net preserves both local and global transcriptomic signals and effectively mitigates over-smoothing. In contrast, RPPA (proteomics) data are relatively low-dimensional and represent downstream protein-level measurements, where biological signals are more compact and graph structures tend to be smoother. Consequently, deeper graph propagation provides limited additional information, leading to comparatively smaller performance gains from the JK-Net mechanism.

Overall, these results demonstrate that JK-Net provides more robust and stable performance across heterogeneous cancer datasets, highlighting the advantage of adaptively integrating multi-scale graph representations for multi-omics cancer subtype prediction.

### 3.2 Visualization

**FIGURE 2:**
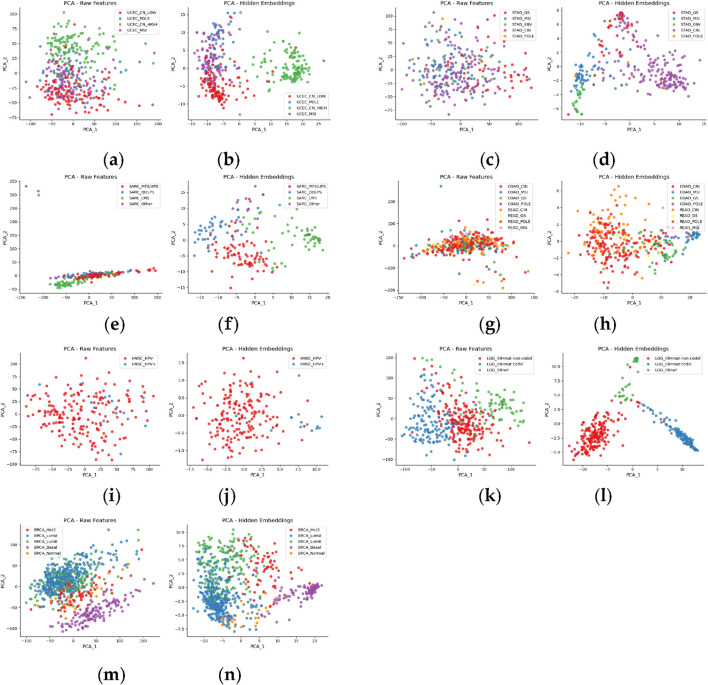
PCA visualization of seven cancer datasets using raw features and MoJKNet embeddings. Each dataset is represented by a pair of subplots: the left panel shows the distribution of Raw Features, and the right panel shows the distribution after representation learning using MoJKNet (MoJKNet embeddings). Specifically, **(a,b)** TCGA-UCEC, **(c,d)** TCGA-STAD, **(e,f)** TCGA-SARC, **(g,h)** TCGA-COADREAD, **(i,j)** TCGA-HNSC, **(k,l)** TCGA-LGG, and **(m,n)** TCGA-BRCA. The x-axis and y-axis correspond to the first and second principal components (PC1 and PC2), respectively. Samples are colored according to their cancer subtypes.

To assess whether the learned embeddings more effectively capture the intrinsic structure of the data, we conducted a visualization analysis comparing the original multi-omics feature matrices (Raw features) with the latent representations generated by our model (MoJKNet embeddings). Principal component analysis (PCA) was applied to project the high-dimensional data into a two-dimensional space. Samples were colored according to their cancer subtypes to evaluate the degree of subtype separability in the projected space. The visualization results for multiple cancer cohorts, including STAD, SARC, LGG, BRCA, UCEC, HNSC, and COADREAD, are presented in Figure 2.

Several key observations can be derived from the visualization analysis. First, the Raw features exhibit substantial overlap across subtypes. In the PCA projections of the original multi-omics feature space, samples from different cancer subtypes are frequently intermingled, with no clear boundaries between groups. This observation reflects the high levels of noise and redundancy inherent in raw multi-omics data, which limit effective subtype separability.

Second, the structure of the embedding space is markedly more distinct. In contrast to the Raw features, the PCA projections of the latent representations (MoJKNet embeddings) display much clearer clustering patterns among different subtypes. For example, in the BRCA cohort, subtype pairs such as LumA versus LumB and Basal versus HER2 form more compact and well-separated clusters compared with their corresponding raw feature distributions. In the LGG cohort, the major molecular classes IDHmut–codel and IDHwt are effectively separated, indicating that the learned embeddings capture key molecular distinctions. Similarly, in the UCEC cohort, the separability between CN_LOW and MSI subtypes is substantially improved in the embedding space.

Third, the learned embeddings demonstrate strong generalization across cancer types. Despite the pronounced heterogeneity among different cancer cohorts, the embedding representations consistently yield clearer subtype structures in most datasets. This consistency suggests that the proposed model effectively extracts cross-omics latent information that enhances subtype discrimination and supports improved downstream predictive performance.

In summary, compared with the Raw features, the embedding representations exhibit markedly stronger subtype discrimination in the visualization space. These results confirm that the proposed model not only reduces noise and redundancy in the original multi-omics data but also captures biologically meaningful latent structures relevant to cancer subtype characterization.

### 3.3 Survival analysis to evaluate MoJKNet embeddings

Survival analysis was conducted separately for the seven cancer datasets using the embeddings learned by the proposed MoJKNet model, following the procedures described in Section 2.3 To further assess the ability of MoJKNet to capture prognostic information, its performance was compared with that of MO-GCAN, a representative graph neural network–based multi-omics integration model. For each cancer type, patients were stratified into high-risk and low-risk groups based on the median risk score, with patients having risk scores above the median assigned to the high-risk group and those with scores below or equal to the median assigned to the low-risk group.

**FIGURE 3:**
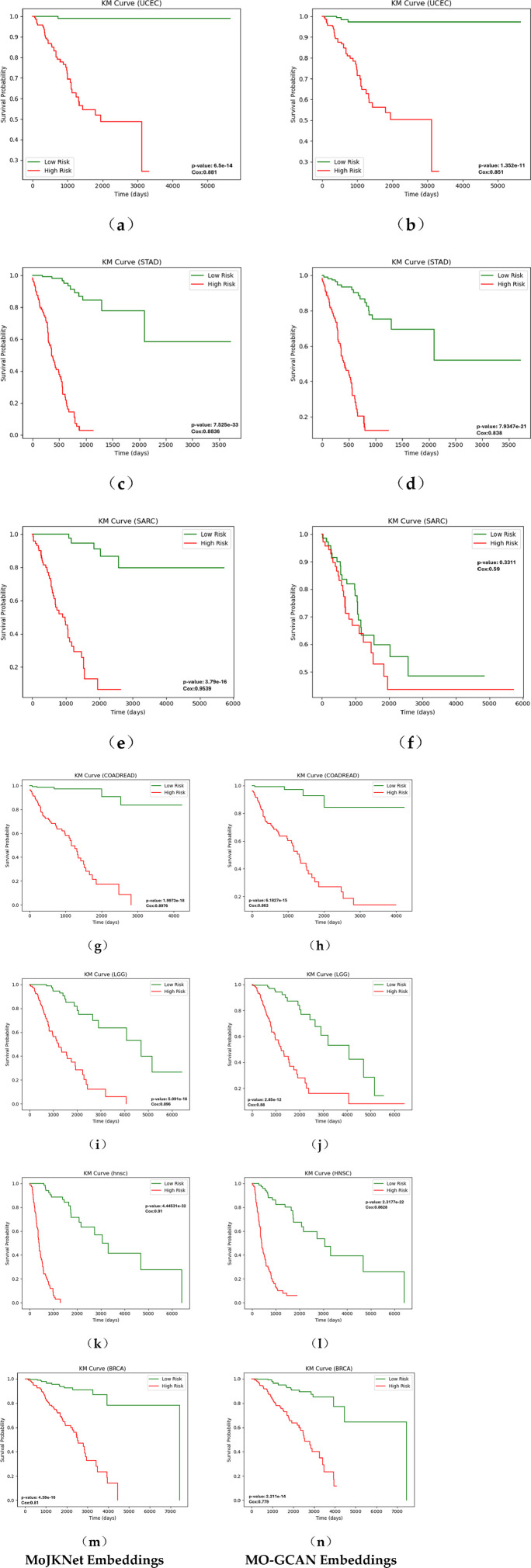
Survival analysis using MoJKNet embeddings and MO-GCAN embeddings across seven cancer datasets. Panels **(a,c,e,g,i,k,m)** display the results obtained using MoJKNet, while panels **(b,d,f,h,j,l,n)** show the corresponding results derived from MO-GCAN. The Kaplan–Meier curves illustrate the proportion of patients in the low-risk and high-risk groups over the follow-up time. Risk groups were determined based on risk scores computed from the embeddings generated by MoJKNet and MO-GCAN, respectively.

The Kaplan–Meier survival curves derived from the embeddings generated by MoJKNet and MO-GCAN are shown in Figure 3. Overall, MoJKNet produces a more pronounced separation between high-risk and low-risk patient groups than MO-GCAN. For example, in the UCEC cohort (Figure 3a), the log-rank test yields a p-value of 6.5 × 10^−14^ when using MoJKNet embeddings, which is substantially smaller than the p-value of 1.32 × 10^−11^ obtained using MO-GCAN embeddings (Figure 3b). These results indicate that the representations learned by MoJKNet more effectively capture prognostic signals associated with patient survival.

In addition to Kaplan–Meier analysis, survival prediction performance was quantitatively evaluated using the concordance index (C-index), which measures the agreement between predicted risk scores and the observed ordering of survival events. A higher C-index indicates stronger predictive discrimination. As illustrated in Figure 3, MoJKNet consistently achieves higher C-index values across most cancer types compared with MO-GCAN, suggesting improved concordance between predicted risks and actual survival outcomes.

Overall, these findings demonstrate that the multi-omics representations learned by MoJKNet not only enhance cancer subtype classification but also preserve richer prognostic information. Consequently, MoJKNet enables more accurate stratification of patient survival risk compared with existing GNN-based multi-omics integration methods.

## 4 Discussion

The proposed MoJKNet model integrates jumping knowledge networks (JK-Net) with an attention-based graph classifier to enable effective utilization of multi-omics features for cancer subtype classification. Experimental results across seven cancer datasets demonstrate that MoJKNet consistently achieves higher macro-averaged precision, recall, and F1-score than existing multi-omics graph-based methods, including MoGCN, MOGONET, and MO-GCAN. These results indicate that MoJKNet more effectively captures latent representations from heterogeneous omics modalities and translates them into improved classification performance.

From a dataset-specific perspective, MoJKNet exhibits clear advantages on cohorts such as TCGA-UCEC, HNSC, COADREAD, and BRCA, particularly in cancer types with relatively balanced sample distributions and distinct subtype-associated molecular patterns. This observation suggests that the multi-scale feature aggregation enabled by the jumping knowledge mechanism, combined with attention-based classification, provides strong representational capacity for modeling complex multimodal molecular information. Furthermore, analysis of feature contribution results (Table 5) reveals that different omics layers contribute unequally to the final classification outcome. Among them, mRNA expression and DNA methylation play dominant roles in subtype discrimination, whereas RPPA data—owing to its lower feature dimensionality and higher noise level—exhibits relatively weaker standalone performance. Nevertheless, RPPA still provides complementary information when integrated with other omics modalities.

**FIGURE 4:**
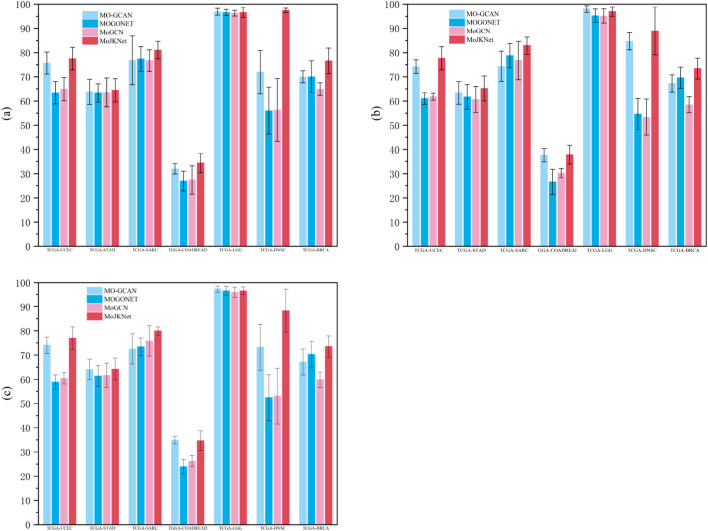
Performance comparison of multi-omics integration models across TCGA cancer datasets. **(a)** Macro-average precision, **(b)** macro-average recall, and **(c)** macro-average F1-score achieved by four models (MO-GCAN, MOGONET, MoGCN, and the proposed MoJKNet). Results are reported as mean ± standard deviation obtained from five-fold cross-validation.

Across the seven TCGA cancer datasets, MoJKNet consistently demonstrates competitive or superior performance compared with existing multi-omics integration models, including MO-GCAN, MOGONET, and MoGCN. As shown in Tables 5–7 and Figure 4, MoJKNet achieves the best results on most datasets in terms of macro-average precision, recall, and F1-score, indicating its effectiveness in capturing discriminative molecular representations for cancer subtype prediction.

In the LGG dataset, MoJKNet achieves classification precision exceeding 96%, with all three subtypes attaining consistently high precision, recall, and F1-scores. This result indicates that the model effectively captures subtype-specific molecular differences at the multi-omics level. As illustrated by the PCA analysis, LGG samples show substantial overlap in the raw multi-omics feature space, whereas the latent embeddings learned by MoJKNet exhibit improved clustering and clearer separation among subtypes. This transformation suggests that the model enhances intrinsic molecular separability during representation learning.

For cancer types such as UCEC, HNSC, and SARC, MoJKNet also demonstrates stable and superior performance compared with baseline models. In particular, on the HNSC dataset, MoJKNet achieves a macro-average precision of 97.46%, substantially outperforming MO-GCAN (71.88%), MOGONET (55.98%), and MoGCN (56.28%). Similar improvements are observed for macro-average recall and F1-score. These cancers are known to exhibit relatively distinct molecular drivers and pathway-level differences, which may facilitate subtype discrimination when multi-omics information is effectively integrated.

BRCA, however, represents a more challenging case due to its pronounced molecular heterogeneity. On the BRCA dataset, MoJKNet achieves an overall precision of approximately 76.54%, outperforming most baseline models but still showing notable variation across different classes. While several cancer subtypes achieve classification accuracies above 80%, the accuracy for the normal tissue group (class 5) remains relatively low (approximately 57%). This discrepancy may be attributed to the inherent molecular differences between cancerous and normal tissues, combined with class imbalance. In particular, subtype 4 contains only 71 samples, which limits the model’s ability to learn robust and representative feature distributions and increases the risk of ambiguous decision boundaries. These observations suggest that for highly heterogeneous cancers such as BRCA, relying solely on molecular omics data may be insufficient. Incorporating additional modalities—such as spatial transcriptomics, immune microenvironment features, or clinicopathological variables—could further improve subtype discrimination.

In the COADREAD dataset, MoJKNet exhibits noticeable improvements over baseline models, achieving higher macro-average precision, recall, and F1-score than MOGONET, MoGCN, and MO-GCAN (Tables 5–7). Nevertheless, the overall predictive performance remains lower than that observed in other cancer types. This limitation is closely related to the high molecular heterogeneity of COADREAD, which contains eight subtypes and poses inherent challenges for classification. PCA-based clustering analysis supports this observation: in the raw feature space, subtype samples are highly intermixed with no clear boundaries, whereas in the fused latent space learned by MoJKNet, samples exhibit improved structural organization. Despite this improvement, certain subtypes, such as CIN and GS, remain partially overlapping because their molecular characteristics are driven primarily by chromosomal copy number alterations rather than transcriptomic or methylation-associated pathway changes. Consequently, the discriminative power of the model for these subtypes remains limited. Nevertheless, these results indicate that the multi-omics autoencoder within MoJKNet effectively captures cross-omics synergistic variation patterns, particularly those associated with immune-related molecular states.

Overall, these findings demonstrate that MoJKNet effectively leverages complementary multi-omics information to enhance cancer subtype classification, particularly in datasets where subtype-specific molecular signals are well defined. Despite these challenges, MoJKNet consistently outperforms baseline models across most cancer types. Moreover, although the attention mechanism in MoJKNet is primarily employed for graph-based classification rather than direct feature fusion, it provides a degree of interpretability by highlighting the relative importance of learned patient representations during subtype prediction. For example, latent features associated with high attention weights may be further explored through functional enrichment analysis to identify candidate biomarkers and subtype-driving mechanisms.

Although MoJKNet introduces additional parameters due to the jumping knowledge architecture and attention-based classifier, the resulting computational overhead remains moderate. The overall computational complexity increases approximately linearly with the number of graph convolution layers, and training remains practical in our experiments, with no significant increase in inference time compared with conventional GCN-based models. Given the substantial performance improvements achieved across multiple cancer types, this trade-off is acceptable for multi-omics cancer subtype classification tasks.

Several limitations of this study should be acknowledged. First, although MoJKNet demonstrates strong performance using molecular-level omics data alone, this design choice inherently constrains the model’s ability to capture phenotypic and contextual heterogeneity that is not fully reflected at the molecular level. Important information related to tumor morphology, spatial organization, immune infiltration, and clinical characteristics is not explicitly modeled, which may limit subtype discrimination for highly heterogeneous cancers such as BRCA and COADREAD. Second, molecular-only representations are more susceptible to class imbalance and sampling bias, particularly for rare subtypes with limited sample sizes. In the absence of complementary clinical or imaging features, the learned representations for underrepresented classes may be less robust, increasing the risk of ambiguous decision boundaries and reduced generalization performance. Third, reliance solely on molecular omics data may restrict the clinical interpretability and translational relevance of the model. While molecular alterations provide critical insights into tumor biology, clinical decision-making often depends on integrated information from pathology, imaging, and patient history. The lack of these modalities may therefore limit the applicability of MoJKNet in real-world clinical settings. Despite these limitations, the modular design of MoJKNet allows for flexible extension. Incorporating additional modalities such as histopathological imaging, spatial transcriptomics, immune microenvironment features, and clinicopathological variables represents a promising direction to improve robustness, interpretability, and predictive power in future work.

Future work will focus on extending MoJKNet toward more comprehensive and clinically relevant multi-modal integration. Incorporating additional data modalities—such as spatial transcriptomics, immune microenvironment features, and clinicopathological variables—could provide complementary phenotypic and contextual information that is not fully captured by molecular omics alone. These modalities may enhance subtype discrimination for highly heterogeneous cancers and improve biological interpretability by linking molecular alterations to spatial organization, immune activity, and clinical outcomes. From a translational perspective, the improvements achieved by MoJKNet highlight its potential utility in precision oncology, particularly for molecular subtype stratification and patient risk assessment. However, several challenges remain for clinical deployment, including the availability and standardization of multi-modal data, the need for improved interpretability of learned representations, and the requirement for robust validation across diverse patient cohorts and sequencing platforms. Addressing these challenges will be essential for translating graph-based multi-omics models such as MoJKNet into real-world clinical decision support systems.

## Data Availability

Publicly available datasets were analyzed in this study. This data can be found here: https://www.kaggle.com/datasets/jiangjielou/the-cbioportal-for-cancer-genomics.
